# Prevalence of Overweight and Obesity and Their Associations With Screen Time and Other Behavioral Factors Among Adolescents in the United Arab Emirates

**DOI:** 10.7759/cureus.117775

**Published:** 2026-10-11

**Authors:** Sana Fatima, Lena Sayed Taha, Sarah Sayed Taha, Ibraheem Mohammed, Abdallah Jaber, Shatha AlSharbatti

**Affiliations:** 1 Hospital-Based Medicine, Dumfries and Galloway Royal Infirmary, Dumfries, GBR; 2 General Practice, Thumbay University Hospital, Ajman, ARE; 3 Internal Medicine, Prime Hospital, Dubai, ARE; 4 Anesthesia, Rashid Hospital, Dubai, ARE; 5 Community Medicine, Gulf Medical University, Ajman, ARE

**Keywords:** childhood obesity, childhood obesity and screen time, childhood obesity in the united arab emirates, childhood overweight, gender and childhood obesity, physical activity, sedentary life-style

## Abstract

Background

Overweight and obesity among adolescents are major public health concerns. Sociodemographic, familial, dietary, and lifestyle factors, including screen time and physical activity, may be associated with excess body weight. In the United Arab Emirates (UAE), limited evidence has examined recreational screen time alongside these factors in the same adolescent population.

Objective

This study aimed to determine the prevalence of overweight and obesity among adolescents aged 14-18 years in the UAE and examine whether greater recreational screen time was associated with higher odds of overweight/obesity. Secondary analyses assessed associations of sociodemographic, familial, dietary, physical activity, and sleep factors with overweight/obesity.

Methodology

A cross-sectional study included 688 adolescents aged 14-18 years recruited from participating high schools and a university teaching hospital in the UAE between December 2023 and April 2024. Height and weight were measured by the investigators, and BMI was classified using sex- and age-specific WHO 2007 growth reference cutoffs. Participants completed a structured questionnaire assessing sociodemographic characteristics, family history, dietary habits, physical activity, screen time, and sleep. Data were analyzed using descriptive statistics, Pearson chi-square tests, and binary logistic regression.

Results

The prevalence of combined overweight/obesity was 39.8% (95% CI: 36.2-43.5%); 27.3% (95% CI: 24.0-30.7%) were overweight, and 12.5% (95% CI: 10.0-15.0%) were obese. Although weekend non-academic screen time was associated with overweight/obesity in the chi-square analysis (p = 0.017), no screen-time category was significant in bivariable logistic regression. In multivariable analysis, overweight/obesity was associated with male sex (AOR = 1.636, 95% CI: 1.155-2.318; p = 0.006), family history of obesity (AOR = 2.014, 95% CI: 1.197-3.389; p = 0.008), frequent eating when not hungry or bored (AOR = 1.884, 95% CI: 1.012-3.509; p = 0.046), and no participation in ≥60 minutes/day of out-of-school moderate-to-vigorous physical activity on any day of the week (AOR = 1.896, 95% CI: 1.285-2.799; p = 0.001).

Conclusions

Overweight and obesity were common among adolescents in this study. Recreational screen time was not associated with overweight/obesity in the regression analysis. Male gender, family history of obesity, frequent eating when not hungry or bored, and no participation in ≥60 minutes/day of out-of-school moderate-to-vigorous physical activity were associated with overweight/obesity after adjustment. Given the cross-sectional design and recruitment from participating schools and a university teaching hospital, these findings represent associations rather than causal relationships and are not nationally representative of UAE adolescents.

## Introduction

The prevalence of overweight and obesity among children and adolescents has increased worldwide. In 2022, more than 390 million children and adolescents aged 5-19 years were overweight, including 160 million with obesity [1]. Overweight during childhood and adolescence is associated with a greater risk and earlier onset of non-communicable diseases, such as type 2 diabetes and cardiovascular disease, and children with obesity are more likely to become adults with obesity [1]. Childhood and adolescent obesity also have adverse psychosocial consequences and are associated with poorer school performance and reduced quality of life [1].

One lifestyle factor that has been linked to this public health problem is increased screen time. As the use of technology has expanded, children and adolescents have spent more time on electronic devices. A recent United Arab Emirates (UAE) study found that daily screen time increased with age, from 3.87 hours per day among children aged seven to 10 years to 5.05 hours per day among adolescents aged 11-15 years [2].

Overweight and obesity are also common among adolescents in the UAE. The 2016 Global School-Based Student Health Survey (GSHS) reported that 39.1% of adolescents aged 13-15 years were overweight and a further 16.2% were obese, while 37.3% of adolescents aged 16-17 years were overweight and a further 17.1% were obese [3]. These findings highlight the need to identify modifiable factors associated with elevated BMI among adolescents in the UAE.

Previous research has shown an association between lifestyle factors, particularly screen time, and BMI among adolescents. For example, a study of 1,517 high school students in suburban Philadelphia found that increased screen time was associated with higher BMI, especially among adolescents at the upper end of the BMI distribution [4]. At the 90th percentile, each unit increase in screen time was associated with a 0.54 kg/m² increase in BMI, compared with 0.18 kg/m² at the 50th percentile [4]. These findings suggest that the association between screen time and BMI may be stronger among adolescents who are already at higher risk of overweight or obesity.

Although a high prevalence of overweight and obesity among adolescents in the UAE has been reported [3], few UAE studies have examined recreational screen time together with behavioral, dietary, physical activity, sleep, and family factors in the same group of adolescents. In addition, recruiting participants only through schools may miss adolescents who use healthcare services but are not reached in school settings. As a result, little is known about how these factors relate to overweight and obesity among adolescents recruited from both school and healthcare settings. Including both settings may provide additional information on factors associated with adolescent overweight and obesity within the study population.

The primary aim of this study was to determine the prevalence of overweight and obesity among adolescents aged 14-18 years in the UAE. The primary hypothesis was that greater recreational screen time would be associated with higher odds of overweight or obesity. Secondary analyses examined the associations of sociodemographic characteristics, family history, dietary behaviors, physical activity, and sleep patterns with overweight or obesity.

## Materials and methods

Conceptual/operational definitions

For the purpose of this study, the conceptual and operational definitions are as follows.

Body mass index (BMI): BMI was calculated by dividing weight in kilograms by height in meters squared (kg/m²) [1].

Overweight: For individuals aged five to 19 years, overweight was defined as a BMI-for-age more than +1 standard deviation above the WHO median and less than +2 standard deviations above the WHO median [5].

Obesity: For individuals aged 5-19 years, obesity was defined as a BMI-for-age more than +2 standard deviations above the WHO median [5].

Overweight/obesity: For statistical analyses, overweight and obesity were combined into a single outcome category, referred to as overweight/obesity. Participants classified as overweight or obese were included in this category, while participants who were underweight or had normal weight were classified as not overweight/obese.

Screen time: Screen time was defined as time spent sitting or lying down in sedentary, screen-based activities, including watching television or using computers, tablets, or mobile devices. Active screen-based activities involving physical movement were excluded in accordance with WHO guidance [6].

Physical activity: Physical activity was defined as participation in moderate-to-vigorous, mostly aerobic physical activity for at least 60 minutes per day. It was assessed according to the number of days per week participants reported engaging in such activity outside school physical education (PE) classes, based on WHO recommendations [6].

Research design

This is a cross-sectional study.

Study population

The study population consisted of adolescents aged 14-18 years recruited from selected private and public high schools and a university teaching hospital in the UAE.

Inclusion criteria

Inclusion criteria included adolescents aged 14-18 years of any nationality or gender who were living in the UAE and were willing to participate. Participants younger than 18 years were required to provide assent and have written informed consent from a parent or legal guardian, whereas participants aged 18 years provided their own written informed consent.

Exclusion criteria

Exclusion criteria were adolescents outside the 14-18-year age range or those who did not provide the required informed consent and assent according to their age.

Sample size calculation

The sample size was calculated using Cochran's formula for estimating a single proportion:

\[ n = \frac{Z^{2} \times P \times (1 - P)}{d^{2}} \] \[ n = \frac{(1.96)^{2} \times 0.20 \times (1 - 0.20)}{(0.03)^{2}} = 682.9 \]

A prevalence (P) of 0.20, a 3% margin of error, and a 95% confidence level were assumed, resulting in a minimum sample size of 683 participants. The assumed prevalence was informed by previously reported data on overweight and obesity among school-aged adolescents in the UAE [7]. A total of 688 participants were recruited and included in the final analysis.

Study settings and participant recruitment

The study was conducted at Thumbay University Hospital and selected high schools in the UAE over a four-month period, from 4 December 2023 to 4 April 2024. Participants were recruited from both school and healthcare settings in order to widen the recruitment base and to include adolescents who might not be reached through school-based recruitment alone. A convenience sampling approach was used; therefore, the sample was not population-based and was not nationally representative of adolescents in the UAE.

Thumbay University Hospital was chosen as a recruitment site because it is the affiliated teaching hospital of the investigators' institution. Adolescents attending the pediatric clinic during the study period were approached together with their accompanying parents or legal guardians. Informed consent was obtained from parents or legal guardians, and assent was obtained from the adolescents before participation.

High schools were contacted by email, and those that agreed to take part were included in the study. School identities are not disclosed in accordance with institutional confidentiality agreements. At participating schools, study information and consent forms were distributed through the school administration to be completed by parents or legal guardians. Students with written parental consent were eligible to participate.

Of the 250 English-medium high schools approached across Dubai, Sharjah, Ajman, Umm Al Quwain, Ras Al Khaimah, and Fujairah, 25 agreed to participate (school participation rate 10%). A total of 600 eligible adolescents aged 14-18 years were recruited from these schools. The number of students approached within the participating schools was not recorded, so an individual-level response rate could not be calculated. A further 88 eligible adolescents were recruited from Thumbay University Hospital. In total, 688 adolescents took part in the study. No participants were excluded, and all 688 were included in the final analysis. The participant flow is summarized in Figure 1.

In both settings, participants completed a structured questionnaire, after which their height and weight were measured by the investigators to calculate BMI.

Data collection instrument

Data were collected using a structured questionnaire developed by the research team based on the study objectives and the relevant literature. The questionnaire covered sociodemographic characteristics, family history, dietary habits, physical activity, screen time, and sleep patterns.

Content and face validity were assessed by a panel of consultant pediatricians. Each expert independently reviewed the questionnaire items for their relevance to the study objectives, clinical appropriateness, clarity, ease of understanding, and suitability for adolescents. The experts also evaluated the response options, the wording, and the overall structure of the questionnaire. The research team then reviewed their feedback, and any items considered unclear, repetitive, or not sufficiently relevant were revised. The complete questionnaire is provided in the Appendices.

Ethical issues

The study commenced following approval from the Gulf Medical University (GMU) Institutional Review Board (IRB) (reference no. IRB-COM-STD-39-MAR-2023; approved on 21 March 2023). The study objectives and procedures were explained to participants and their parents or legal guardians, and written informed consent was obtained from the legal guardians of participants younger than 18 years prior to participation. Assent was also obtained from the adolescents after the study was explained in age-appropriate language. Adolescents were informed that participation was voluntary and that they could withdraw at any time without consequences. Confidentiality and anonymity were ensured, and access to the data was limited to the research team. Data will be stored for at least three years in accordance with GMU policy.

Methodology

Official approval was obtained from all participating sites before study initiation. Participants completed the structured questionnaire, after which anthropometric measurements were obtained using standardized procedures [5]. Weight was measured using a calibrated digital weighing scale, with participants wearing light clothing and no footwear, and recorded to the nearest 0.1 kg. Height was measured using a stadiometer, with participants standing barefoot in an upright position, and recorded to the nearest 0.1 cm. BMI was calculated as weight in kilograms divided by height in meters squared (kg/m²).

Participants were classified according to sex- and age-specific BMI-for-age cutoff values from the WHO 2007 growth reference [5]. Each participant’s BMI was compared with the corresponding WHO cutoff values at -2, +1, and +2 SD to classify them as underweight, normal weight, overweight, or obese. Overweight and obesity were subsequently combined for the analysis.

Eating when not hungry or feeling bored was assessed using the questionnaire item, “Do you eat when you are not hungry or feeling bored?” Responses were categorized as never, rarely, sometimes, or frequently.

Physical activity was assessed using a questionnaire item asking participants how many days per week they engaged in at least 60 minutes per day of moderate-to-vigorous, mostly aerobic physical activity outside school PE classes.

Details of data storage and analysis

Data were entered into Microsoft Excel (Microsoft® Corp., Redmond, WA) and analyzed using IBM SPSS Statistics version 29 (IBM Corp., Armonk, NY). Descriptive statistics were used to summarize participant characteristics. Associations between categorical variables were assessed using the Pearson chi-square test.

No statistical imputation was performed for missing values. For descriptive and bivariate analyses, an available-case approach was used: participants with a missing response for a particular variable were excluded only from analyses involving that variable, and percentages were calculated using the number of valid responses as the denominator. Consequently, denominators may vary slightly across variables and are reported in the corresponding tables. For multivariable logistic regression, a complete-case approach was used, whereby participants with missing data for the outcome or any variable included in the model were excluded from that analysis.

Binary logistic regression was used to examine factors associated with overweight/obesity. The outcome was dichotomized as overweight/obesity versus not overweight/obese. Bivariable logistic regression was initially performed to estimate crude odds ratios (CORs) with 95% confidence intervals (CIs). Variables with p < 0.05 in the bivariable logistic regression analysis were considered for inclusion in the multivariable logistic regression model to estimate adjusted odds ratios (AORs) with 95% CIs.

The variables considered for regression analysis included gender, weekend non-academic screen time, family history of obesity, fast-food consumption, eating when not hungry or bored, water intake, and physical activity. Water intake was evaluated during model development but was not retained in the final multivariable model.

For logistic regression, physical activity was dichotomized as participation in moderate-to-vigorous physical activity for at least 60 minutes per day on at least one day per week versus no participation on any day of the week. Accordingly, participants reporting no such activity on any day of the week were classified as physically inactive for the regression analysis. This operational definition was based on the questionnaire response categories and was not intended to represent full adherence to the WHO recommendation of at least 60 minutes of moderate-to-vigorous physical activity daily [6].

Fast-food consumption was dichotomized as more than five times per week versus five times or fewer. Categorical variables were entered using appropriate reference categories.

Water intake was significant in the bivariable analysis but was not retained in the final multivariable model. This decision was made during model development and was not prespecified; therefore, the adjusted estimates should be interpreted with appropriate caution. Weekend non-academic screen time, the primary exposure of interest, was not entered into the multivariable model because no category was significantly associated with overweight/obesity in the bivariable logistic regression analysis.

Statistical significance was defined as p < 0.05. Questionnaire responses were numerically coded for statistical analysis, and categorical variables were grouped according to the questionnaire response options and predefined analytical categories. For logistic regression, weekend non-academic screen time was categorized as none, ≤1 hour, two to three hours, four to five hours, and >5 hours, with none used as the reference category.

## Results

Figure 1 illustrates the participant flow. A total of 250 English-medium high schools were approached, of which 25 agreed to participate. Six hundred eligible adolescents were recruited from the participating schools, and a further 88 eligible adolescents were recruited from Thumbay University Hospital, giving a total of 688 participants. No participants were excluded, and all 688 participants were included in the final analysis. 

**Figure 1:**
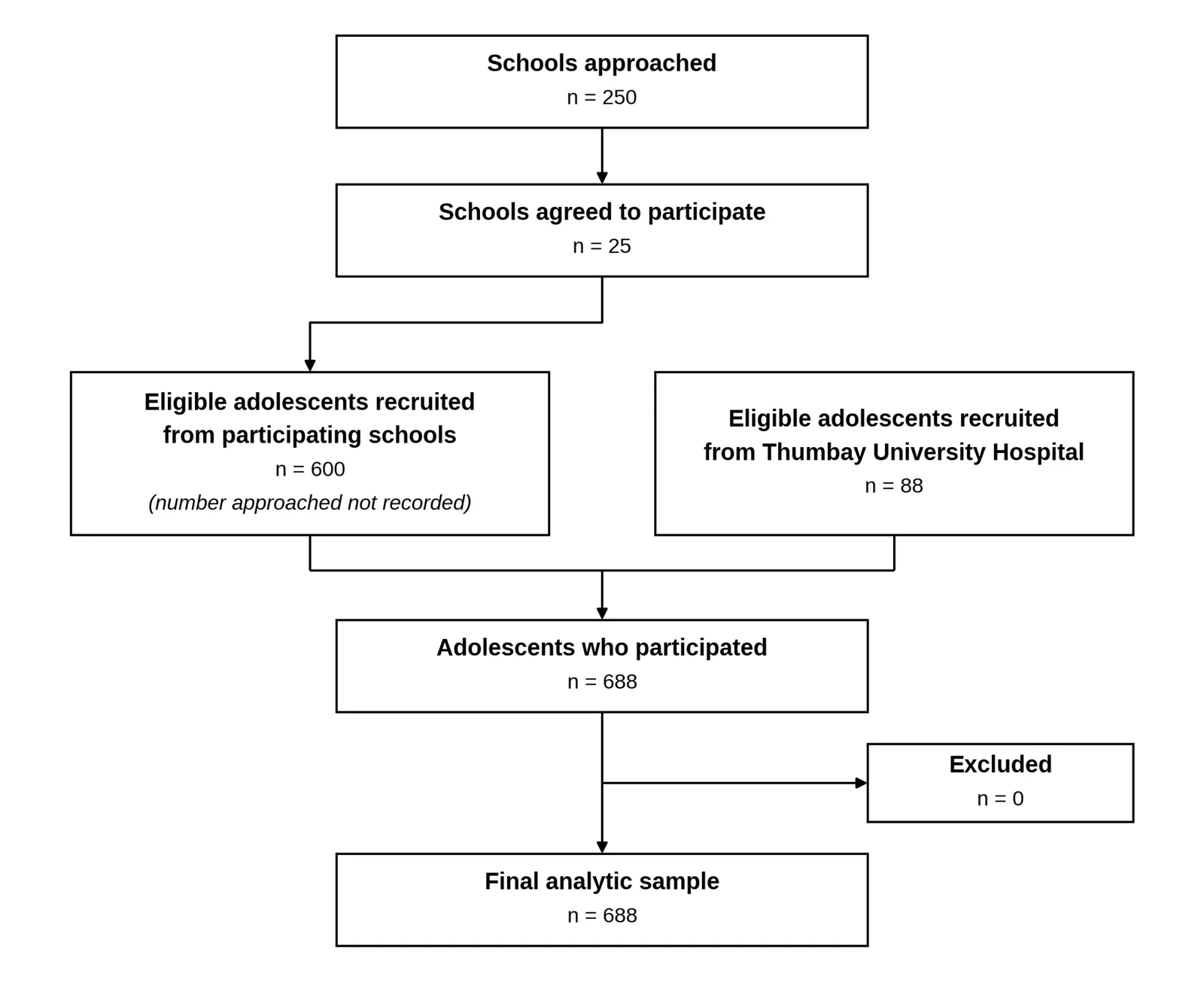
Participant Flow Diagram

Figure 2 illustrates the weight-status distribution of the 688 adolescents included in the study. The combined prevalence of overweight or obesity was 39.8% (95% CI: 36.2-43.5%). The prevalence of overweight was 27.3% (95% CI: 24.0-30.7%), while the prevalence of obesity was 12.5% (95% CI: 10.0-15.0%). Of the remaining participants, 15 (2.2%) were underweight, and 399 (58.0%) had normal weight. 

**Figure 2:**
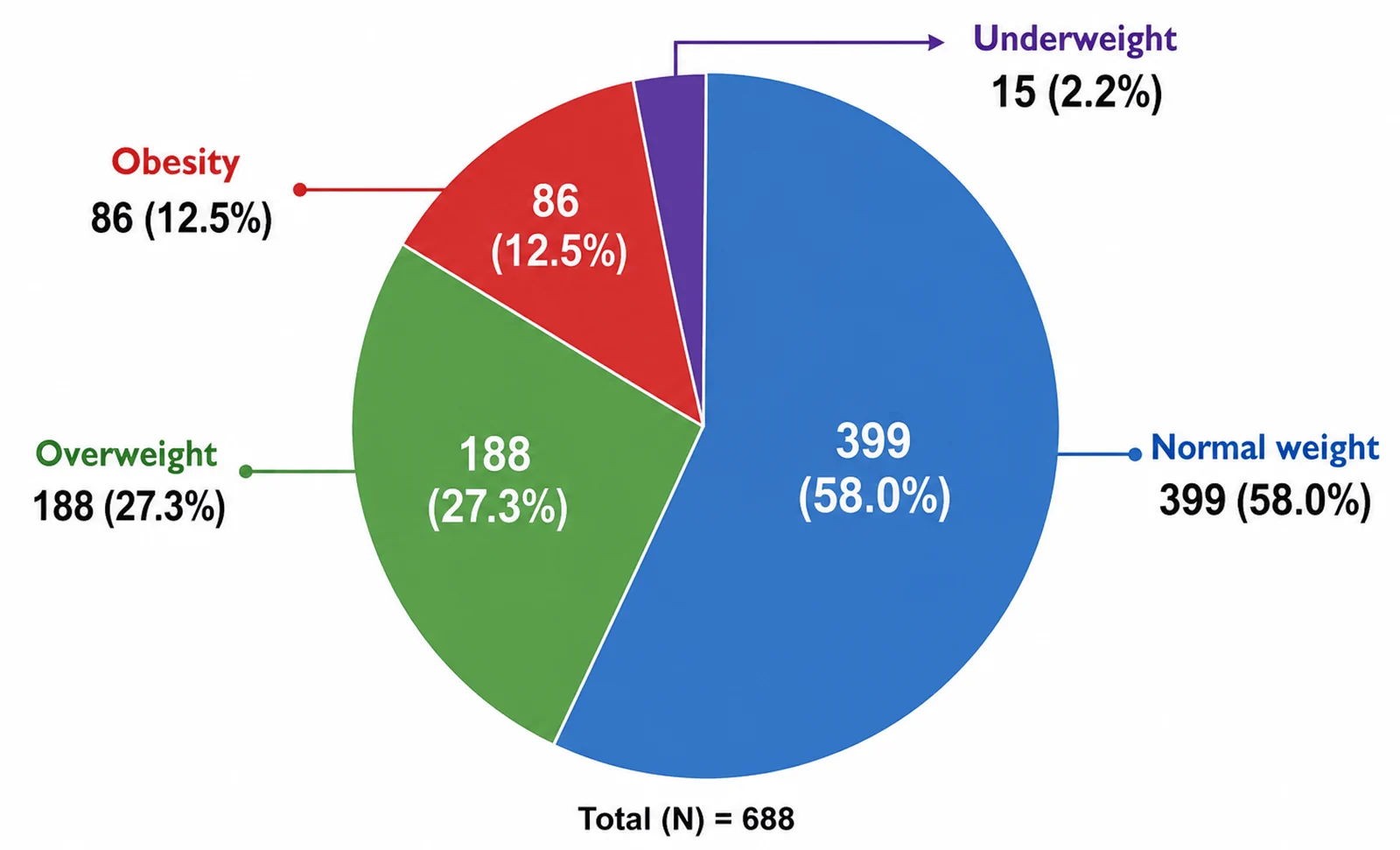
Weight-Status Distribution Among Study Participants (N = 688)

Table 1 presents the association between sociodemographic characteristics and overweight or obesity. A total of 688 adolescents participated in the study, and all 688 participants were included in the final analysis. No participants were excluded. The prevalence of overweight and obesity varied by age, with the highest rate observed among 17-year-olds (45.4%) and the lowest among 18-year-olds (32.6%); however, this difference was not statistically significant (p = 0.379). A significant association was observed with gender, with a higher prevalence among males than females (47.3% vs. 34.9%, p = 0.001). No significant difference was found according to school type (p = 0.642). 

**Table 1:** Prevalence of Overweight and Obesity by Age, Gender, and School Type

	Subcategory	Not overweight/obese		Overweight/obese		p-value	Pearson chi-square
		n	%	n	%		
Age (in years)	14	121	59.9	81	40.1	0.379	4.204
	15	71	62.8	42	37.2		
	16	81	60.0	54	40.0		
	17	83	54.6	69	45.4		
	18	58	67.4	28	32.6		
Gender	Female	269	65.1	144	34.9	0.001	10.602
	Male	145	52.7	130	47.3		
School	Private school	396	60.4	260	39.6	0.642	0.216
	Public school	18	56.3	14	43.8		

Table 2 presents the association between screen time behaviors and overweight or obesity among the study participants. Most screen time behaviors were not significantly associated with overweight or obesity. School-related screen time was not significantly associated with overweight or obesity (p = 0.190). Although the highest prevalence was observed among participants reporting no school-related screen time (66.7%), this subgroup was very small (n = 6). In contrast, weekend non-academic screen time was significantly associated with overweight or obesity (p = 0.017). No significant associations were found for parental screen restrictions, electronic device use with friends, or frequency of social media use.

**Table 2:** Association Between Screen Time Behaviors and Overweight or Obesity Percentages are calculated among participants with available responses; missing responses were excluded from the respective analysis.

Screen time practices	Subcategory	Not overweight/obese		Overweight/obese		p-value	Pearson chi-square
		n	%	n	%		
Screen time (hours) spent on school-related activities	None	3	33.3	6	66.7	0.190	7.446
	≤ 1	35	54.7	29	45.3		
	2-3	76	61.8	47	38.2		
	4-5	29	51.8	27	48.2		
	>5	39	55.7	31	44.3		
Screen time (hours) spent on nonactive or non-academic purposes on a school day	None	9	47.4	10	52.6	0.503	3.337
	≤ 1	64	56.6	49	43.4		
	2-3	145	61.7	90	38.3		
	4-5	126	63.3	73	36.7		
	>5	70	57.4	52	42.6		
Screen time (hours) spent on nonactive or non-academic purposes on a weekend day	None	5	41.7	7	58.3	0.017	12.034
	≤1	32	62.7	19	37.3		
	2-3	91	63.2	53	36.8		
	4-5	131	67.9	62	32.1		
	>5	155	53.8	133	46.2		
Whether parents/ guardians of participants limit their non-educational screen time	Yes	171	59.6	116	40.4	0.788	.072
	No	243	60.6	158	39.4		
Hours per day spent engaged with a friend on electronic devices (i.e., for play or communicating)	None	61	70.1	26	29.9	0.333	4.579
	≤ 1	125	59.5	85	40.5		
	2-3	125	58.1	90	41.9		
	4-5	39	55.7	31	44.3		
	>5	64	60.4	42	39.6		
How often participants used social media	I don’t use social media	29	70.7	12	29.3	0.132	11.158
	A few times a month	14	77.8	4	22.2		
	About once a week	11	61.1	7	38.9		
	A few times a week	23	59.0	16	41.0		
	About once a day	48	69.6	21	30.4		
	Several times a day	155	59.6	105	40.4		
	About once an hour	59	60.2	39	39.8		

Table 3 presents the association between personal and family medical history and overweight or obesity. A personal history of chronic diseases or allergies was not significantly associated with overweight or obesity. Among family history factors, a family history of obesity was significantly associated with a higher prevalence of overweight or obesity (53.2% vs. 38.1%, p = 0.011). Family histories of hypertension and diabetes were also associated with a slightly higher prevalence, but these differences were not statistically significant (p = 0.076 and p = 0.168, respectively).

**Table 3:** Association Between Personal and Family Medical History and Overweight or Obesity

Variable	Subcategory	Not overweight/obese		Overweight/obese		p-value	Pearson chi-square
		N	%	N	%		
Personal history							
Chronic diseases	Yes	24	57.1	18	42.9	0.679	0.172
	No	390	60.4	256	39.6		
Allergies	Yes	50	57.5	37	42.5	0.582	0.304
	No	364	60.6	237	39.4		
Family history							
Diabetes mellitus	Yes	97	55.7	77	44.3	0.168	1.905
	No	317	61.7	197	38.3		
Hypertension	Yes	51	52.0	47	48.0	0.076	3.155
	No	363	61.5	227	38.5		
High cholesterol	Yes	81	56.3	63	43.8	0.324	1.170
	No	333	61.2	211	38.8		
Hypothyroidism	Yes	21	65.6	11	34.4	0.519	0.416
	No	393	59.9	263	40.1		
Chronic heart disease	Yes	28	66.7	14	33.3	0.375	0.787
	No	386	59.8	260	40.2		
Asthma	Yes	47	56.0	37	44.0	0.399	0.712
	No	367	60.8	237	39.2		
Stroke	Yes	8	57.1	6	42.9	0.815	0.055
	No	406	60.2	268	39.8		
Liver disease	Yes	10	52.6	9	47.4	0.496	0.464
	No	404	60.4	265	39.6		
Obesity	Yes	36	46.8	41	53.2	0.011	6.517
	No	378	61.9	233	38.1		
Sleep apnea	Yes	14	66.7	7	33.3	0.537	0.381
	No	400	60.0	267	40.0		
None of the above	Yes	206	62.8	122	37.2	0.179	1.810
	No	208	57.8	152	42.2		

Table 4 summarizes the association between dietary habits, eating behaviors, and overweight or obesity. Higher prevalence rates were observed among participants who consumed four meals per day and those with low fruit and vegetable intake; however, these differences were not statistically significant. Significant associations were identified for water intake (p = 0.004) and eating when not hungry or feeling bored (p = 0.037). Other dietary factors, including fast-food and snack consumption, were not significantly associated with overweight or obesity.

**Table 4:** Association Between Dietary Habits and Overweight or Obesity Percentages are calculated among participants with available responses; missing responses are excluded from the respective analysis.

Dietary habits	Subcategory	Not overweight/obese		Overweight/obese		p-value	Pearson chi-square
		n	%	n	%		
Number of meals consumed per day	1	16	69.6	7	30.4	0.595	2.783
	2	107	58.2	77	41.8		
	3	239	61.4	150	38.6		
	4	45	54.9	37	45.1		
	≥5	7	70	3	30		
Number of servings of fruit and vegetables consumed per week	None	22	51.2	21	48.8	0.604	1.852
	1	85	61.2	54	38.8		
	2	136	59.4	93	40.6		
	≥3	171	61.7	106	38.3		
Number of sweetened beverages (i.e., soda) consumed per week	None	118	68.2	55	31.8	0.091	6.469
	1	126	58.6	89	41.4		
	2	95	57.2	71	42.8		
	≥3	75	56	59	44		
Water intake in liters per day	0.5	105	67.7	50	32.3	0.004	13.344
	1	150	63.6	86	36.4		
	1.5	100	58.1	72	41.9		
	>1.5	59	47.6	65	52.4		
Number of servings of snacks per week	None	20	62.5	12	37.5	0.209	4.536
	1	93	58.1	67	41.9		
	2	139	65.9	72	34.1		
	≥3	162	56.8	123	43.2		
Fast food intake per week	None	58	61.7	36	38.3	0.132	7.08
	1	186	60.6	121	39.4		
	2	119	60.4	78	39.6		
	3-5	46	63.0	27	37.0		
	>5	5	29.4	12	70.6		
On average, how often participants consumed food when not hungry or feeling bored	Never	61	68.5	28	31.5	0.037	8.511
	Rarely	116	65.9	60	34.1		
	Sometimes	164	57.1	123	42.9		
	Frequently	73	53.7	63	46.3		

Table 5 presents the association between school food sources, eating habits at school, and overweight or obesity. No significant associations were observed between cafeteria use or packed lunch consumption and overweight or obesity, although slight differences in prevalence were noted.

**Table 5:** Association Between School Food Sources and Overweight or Obesity

Source of food consumed at school	Subcategory	Not overweight/obese		Overweight/obese		p-value	Pearson chi-square
		n	%	n	%		
How often participants ate from the school cafeteria	Every day	57	61.3	36	38.7	0.384	4.162
	Once a week	97	59.9	65	40.1		
	2 times a week	30	51.7	28	48.3		
	3 times a week	34	53.1	30	46.9		
	Never	196	63	115	37		
How often participants brought packed lunches to school	Every day	212	61.3	134	38.7	0.626	2.604
	Once a week	29	67.4	14	32.6		
	2 times a week	23	63.9	13	36.1		
	3 times a week	38	59.4	26	40.6		
	Never	112	56.3	87	43.7		

Table 6 presents the association between physical activity, PE participation, sleep patterns, and overweight or obesity. No statistically significant associations were found in the chi-square analyses between these factors and overweight or obesity. Among participants with available responses, the highest prevalence of overweight or obesity was observed among those who did not engage in physical activity (46.9%) and those who did not participate in PE (48.6%). Regarding weekend sleep duration, the highest prevalence was observed among participants who slept 10 hours or more (44.7%), while the lowest was observed among those who slept six hours or less (37.1%). Similarly, on school nights, the lowest prevalence was reported among participants who slept nine to 10 hours (35.8%); however, no statistically significant association was identified.

**Table 6:** Association Between Physical Activity, Sleep Patterns, and Overweight or Obesity

Variable	Subcategory	Not overweight/obese		Overweight/ obese		p-value	Pearson chi-square
		n	%	n	%		
Number of days per week participants engaged in out-of-school moderate-to-vigorous physical activity (≥60 minutes/day)	None	94	53.1	83	46.9	0.132	7.079
	1 day	105	67.3	51	32.7		
	2 days	76	60.8	49	39.2		
	3 days	59	59.6	40	40.4		
	≥4 days	80	61.1	51	38.9		
Number of days per week participants engaged in physical education (PE) classes	0	37	51.4	35	48.6	0.186	7.496
	1	266	64.1	149	35.9		
	2	79	54.5	66	45.5		
	3	11	52.4	10	47.6		
	4	8	61.5	5	38.5		
	5	13	59.1	9	40.9		
Number of hours participants slept per day on weekends	>10	47	55.3	38	44.7	0.673	1.541
	9-10	125	59	87	41		
	7-8	154	61.4	97	38.6		
	≤6	88	62.9	52	37.1		
Number of hours participants slept per day on school nights	>10	7	58.3	5	41.7	0.842	0.830
	9-10	43	64.2	24	35.8		
	7-8	160	61.1	102	38.9		
	≤6	204	58.8	143	41.2		

Table 7 presents the results of the bivariable and multivariable logistic regression analyses of factors associated with overweight or obesity among adolescents. CORs, AORs, and 95% CIs are reported. Variables that were statistically significant in the bivariable analysis were considered for inclusion in the multivariable logistic regression model. Water intake was evaluated during model development but was not retained in the final multivariable model. Statistical significance was set at p < 0.05.

**Table 7:** Bivariable and Multivariable Logistic Regression Analysis of Factors Associated With Overweight or Obesity Reference categories are indicated by “Ref.” “Not included” indicates variables that were not retained in the final multivariable model. Statistical significance was defined as p < 0.05. AOR, adjusted odds ratio; CI, confidence interval; COR, crude odds ratio

Variable	Subcategory	COR	95% CI	p-value	AOR	95% CI	p-value
Gender	Female (Ref.)	1.00	-	-	-	-	-
	Male	1.675	1.227–2.287	0.001	1.636	1.155–2.318	0.006
Screen time (hours) spent on nonactive, non-academic purposes on a weekend day	None (Ref.)	1.00	-	-	Not included	-	-
	≤1	0.424	0.118–1.526	0.189	Not included	-	-
	2–3	0.416	0.126–1.376	0.151	Not included	-	-
	4–5	0.338	0.103–1.108	0.073	Not included	-	-
	> 5	0.613	0.190–1.976	0.412	Not included	-	-
Family history of obesity	No (Ref.)	1.00	-	-	-	-	-
	Yes	1.848	1.147–2.975	0.012	2.014	1.197–3.389	0.008
Fast-food consumption per week	≤5 (Ref.)	1.00	-	-	-	-	-
	>5	3.747	1.305–10.757	0.013	2.757	0.929–8.181	0.068
Habit of consuming food when not hungry or feeling bored	Never (Ref.)	1.00	-	-	-	-	-
	Rarely	1.127	0.653–1.944	0.668	1.105	0.602–2.030	0.747
	Sometimes	1.634	0.986–2.707	0.057	1.543	0.883–2.696	0.127
	Frequently	1.880	1.074–3.292	0.027	1.884	1.012–3.509	0.046
Water intake (L/day)	0.5 (Ref.)	1.00	-	-	Not included	-	-
	1	1.204	0.784–1.848	0.396	Not included	-	-
	1.5	1.512	0.961–2.378	0.074	Not included	-	-
	>1.5	2.314	1.421–3.767	<0.001	Not included	-	-
Participation in out-of-school moderate-to-vigorous physical activity for at least 60 minutes per day	Yes (Ref.)	1.00	-	-	-	-	-
	No	1.479	1.047–2.090	0.026	1.896	1.285–2.799	0.001

The bivariable logistic regression analysis demonstrated that male adolescents (COR = 1.675, 95% CI: 1.227-2.287, p = 0.001), a family history of obesity (COR = 1.848, 95% CI: 1.147-2.975, p = 0.012), frequent eating when not hungry or feeling bored (COR = 1.880, 95% CI: 1.074-3.292, p = 0.027), frequent fast-food consumption (COR = 3.747, 95% CI: 1.305-10.757, p = 0.013), and physical inactivity (defined as no participation in moderate-to-vigorous physical activity for at least 60 minutes per day on any day of the week) (COR = 1.479, 95% CI: 1.047-2.090, p = 0.026) were significantly associated with overweight or obesity. In the multivariable logistic regression model, male gender (AOR = 1.636, 95% CI: 1.155-2.318; p = 0.006), a family history of obesity (AOR = 2.014, 95% CI: 1.197-3.389; p = 0.008), frequent eating when not hungry or feeling bored (AOR = 1.884, 95% CI: 1.012-3.509; p = 0.046), and physical inactivity (AOR = 1.896, 95% CI: 1.285-2.799; p = 0.001) remained significantly associated with overweight or obesity. Frequent fast-food consumption was no longer statistically significant in the multivariable model (AOR = 2.757, 95% CI: 0.929-8.181; p = 0.068).

## Discussion

In this study, 39.8% of adolescents were overweight or obese (27.3% overweight and 12.5% obese). This was lower than the prevalence reported in the 2016 GSHS, which found that 55.3% of UAE adolescents aged 13-15 years were overweight or obese (39.1% overweight and 16.2% obese) [3], but slightly higher than the 34.7% reported by Baniissa et al. among school-aged adolescents in the UAE [8]. Differences in age groups, settings, and methods limit direct comparison. In the multivariable analysis, male gender, a family history of obesity, frequently eating when not hungry or bored, and reporting no participation in at least 60 minutes of out-of-school moderate-to-vigorous physical activity on any day of the week were significantly associated with overweight/obesity. Recreational screen time, the primary exposure, was not associated with overweight/obesity in the regression analysis.

Factors related to overweight and obesity in adolescents

Gender

In this study, gender was significantly associated with overweight and obesity (p = 0.001). Overall, the prevalence of overweight or obesity was significantly higher among males (n = 130, 47.3%) than females (n = 144, 34.9%). After adjustment for other variables included in the multivariable model, male gender remained significantly associated with overweight or obesity (AOR = 1.636, 95% CI: 1.155-2.318; p = 0.006). This finding is consistent with previous studies reporting a greater prevalence of overweight and obesity among boys than girls.

For example, the Youth Risk Behavior Survey in the United States reported obesity rates of 16.8% among high school males compared with 10.8% among females [9]. Similar findings have been reported in the Middle East and North Africa (MENA) region. A systematic review of 21 studies found childhood overweight and obesity prevalence of up to 49.4%, depending on the diagnostic criteria used, with male adolescents consistently identified as a risk factor [10]. Likewise, a retrospective study from Saudi Arabia involving 351,195 children aged two to 19 years reported a higher prevalence of obesity among boys (10.4%) than among girls (8.3%) [11].

Similar results have also been reported in the UAE. A study conducted in Dubai in 2021 among children aged three to 12 years attending primary healthcare centers during the COVID-19 pandemic found an overall obesity prevalence of 35.8%, with a significantly higher prevalence among males than females (p = 0.012) [12].

Family History

Adolescents with a family history of obesity had significantly higher odds of being overweight or obese. Among those with a family history of obesity, 53.2% (n = 41) were classified as overweight or obese (p = 0.011). A family history of obesity was associated with higher odds of overweight or obesity (AOR = 2.014, 95% CI: 1.197-3.389; p = 0.008). This finding is consistent with previous research. A study conducted in Tabuk City, Saudi Arabia, reported that adolescents with a family history of obesity were 5.735 times more likely to be obese (95% CI: 3.318-9.912; p < 0.001) [13]. Similarly, a review of 12 Iranian studies involving high school students found a significant association between overweight, obesity, family history, and other related factors [14].

Family history of diabetes and hypertension showed higher prevalences of overweight or obesity in some categories, but neither association was statistically significant. These findings were therefore not considered significant associations in the present study and are not interpreted further. Nevertheless, a Swedish study of 11,050 children found that overweight or obesity was more common among children whose parents had diabetes (31% vs. 21%) [15].

Poor Dietary Habits

This study found that participants who consumed four meals per day had the highest prevalence of overweight or obesity (n = 37, 45.1%). In contrast, the lowest prevalence was observed among those who consumed five or more meals per day (30.0%, n = 3) or one meal per day (30.4%, n = 7). However, meal frequency was not significantly associated with overweight or obesity (p = 0.595). These findings are consistent with those of a 2023 prospective study conducted in Spain, which followed 379 children for 15 months and found no significant association between consuming three to five meals per day and BMI or abdominal obesity [16].

In contrast, eating behavior showed a stronger association with weight status. Participants who frequently ate when not hungry or feeling bored had the highest prevalence of overweight or obesity (n = 63, 46.3%), and this association was statistically significant (p = 0.037). In the multivariable analysis, frequent eating when not hungry or feeling bored was associated with higher odds of overweight or obesity (AOR = 1.884, 95% CI: 1.012-3.509; p = 0.046). Similar findings were reported in a 2023 UK laboratory study, in which young children ate more after a boredom mood induction than in a neutral mood, particularly when their parents often used food to comfort them [17]. Although that study involved younger children and used a different methodology, its findings provide some support for a possible relationship between boredom-related eating and increased food intake.

Sedentary Lifestyle

In our study, 25.7% of participants (n = 177) reported no participation in at least 60 minutes of moderate-to-vigorous physical activity on any day of the week outside school PE classes. In comparison, 19.0% (n = 131) reported participating in this level of physical activity on at least four days per week. In the multivariable analysis, participants who reported no such physical activity on any day of the week had higher odds of overweight or obesity than those who reported participation on at least one day per week (AOR = 1.896, 95% CI: 1.285-2.799; p = 0.001). These findings are consistent with a 2023 South African study, which reported that children who did not meet the recommended 60 minutes of daily moderate-to-vigorous physical activity were more likely to be overweight or obese [18]. Similarly, a 2017 review identified insufficient physical activity as an important factor associated with childhood and adolescent obesity in the MENA region, along with other lifestyle factors such as excessive screen time and frequent consumption of fried foods [19].

Most students in our study (60.3%) attended only one PE session per week. Although PE frequency was not significantly associated with overweight or obesity in the present study, this finding provides additional context regarding school-based physical activity among the participants. The WHO recommends that adolescents engage in an average of at least 60 minutes of moderate-to-vigorous physical activity per day across the week [6]. In the UAE study by Baniissa et al., which included 932 adolescents, physically inactive students were approximately twice as likely to be obese (AOR = 2.09, 95% CI: 1.36-3.22) [8]. In the present study, reporting no participation in at least 60 minutes of out-of-school moderate-to-vigorous physical activity on any day of the week was associated with higher odds of overweight or obesity in the multivariable analysis.

Similar findings have been reported in Jordan, where a 2024 study of 600 adolescents identified lack of resources as the main barrier to physical activity. Students reported limited access to sports facilities, a lack of exercise equipment at home, and insufficient time because of busy school schedules [20]. Although PE is mandatory in UAE schools, the amount of PE provided varies between schools, with only 79% delivering at least 100 minutes per week. Physical activity participation also appeared to decline during the secondary-school years, decreasing from 36% among children aged 10-13 years to 28% among adolescents aged 14-18 years [21]. These findings provide context for the physical inactivity observed in our study and suggest that school and environmental factors may influence opportunities for physical activity among adolescents.

Sleep Patterns

In our study, 50.4% of participants reported sleeping six hours or less on school nights, below the recommended eight to 10 hours for adolescents [22]. However, sleep duration was not significantly associated with overweight/obesity in the present study (p = 0.842). Although previous studies, in Riyadh, for example, have reported associations between short sleep duration and obesity [23], differences in study populations and methods may account for the inconsistent findings. Further studies using objective or validated measures of sleep duration and quality may help clarify this relationship among UAE adolescents.

Screen Time

Our study found no significant association between school-related screen time or weekday non-academic screen time and overweight or obesity (p = 0.190 and p = 0.503, respectively). Weekend non-academic screen time was significantly associated with overweight or obesity in the unadjusted analysis (p = 0.017). However, the pattern across categories was not consistent: the highest prevalence was found among participants with no weekend screen time (58.3%, n = 12), and the lowest among those with four to five hours per day (32.1%). In the bivariable logistic regression analysis, no weekend screen-time category was significantly associated with overweight or obesity, and the variable was therefore not included in the multivariable model. Therefore, the present study does not provide evidence of an adjusted association between weekend recreational screen time and overweight/obesity.

In contrast, several previous studies have reported positive associations. A cross-sectional study conducted in the Makkah region of Saudi Arabia involving 450 children and adolescents found that greater use of electronic devices, including mobile phones, tablets, televisions, and computers, was associated with higher BMI [24]. The study also reported that increased screen time was linked to shorter sleep duration, longer time to fall asleep, and longer time spent in bed. Likewise, a school-based cross-sectional study of 550 adolescents in Addis Ababa, Ethiopia, found that more than half of the participants exceeded the recommended daily screen time. Higher screen time, together with eating while watching television and lower physical activity, was significantly associated with overweight and obesity [25].

Social media use was also examined as a specific form of recreational screen time.

Social Media

In our study, the prevalence of overweight and obesity did not follow a clear pattern across levels of social media use (range: 22.2%-41.0%), and the association was not statistically significant (p = 0.132).

Similar findings have been reported previously. For example, a survey of 1,184 adolescents, including 563 boys and 621 girls, found that 82.5% exceeded the recommended screen time limit of two hours per day. Despite the high level of screen use, the study found no significant association between screen time and either general or abdominal obesity [26]. 

Strengths and limitations

This study has several strengths. It included adolescents aged 14-18 years, with a final sample of 688 participants exceeding the calculated minimum sample size. Participants were recruited from 25 schools across six emirates and a university teaching hospital, providing participants from different educational and community settings. Height and weight were measured directly by the investigators using standardized procedures, and BMI for age was classified using the WHO 2007 growth reference. The study examined a broad range of behavioral and familial factors, including recreational screen time, physical activity, dietary behaviors, sleep, and family history, and used multivariable logistic regression to examine factors associated with the combined outcome of overweight/obesity. The findings also provide additional UAE-specific evidence and were considered alongside existing UAE and regional literature.

Several limitations should be considered. First, the cross-sectional design does not allow temporal or causal relationships to be established. Participants were recruited from schools that agreed to participate and from a university teaching hospital, and the schools were English-medium. Most participants were recruited from private schools; therefore, the sample is not nationally representative of adolescents in the UAE, and selection bias is possible.

Second, several behavioral and family-history variables were self-reported and may be subject to recall or social desirability bias. The study-specific questionnaire was reviewed by consultant pediatricians for content validity, relevance, and clarity but was not formally assessed using psychometric reliability testing. The item assessing eating when not hungry or feeling bored was also a single questionnaire item rather than a validated measure of emotional or boredom-related eating. These factors may have introduced measurement error in the assessment of behavioral exposures.

Third, variables for the multivariable model were considered based on statistical significance in bivariable analyses rather than a fully prespecified causal framework. Although consistent with the exploratory objectives, this approach may introduce model-selection bias and residual confounding. Age was not included in the final model, and clustering of participants within schools was not accounted for. Formal assessment of model fit and influential observations was not performed. Multiple behavioral and familial variables were also examined, increasing the possibility of chance findings. The association with frequent eating when not hungry or bored was close to the conventional significance threshold (p = 0.046) and should therefore be interpreted cautiously.

Finally, some questionnaire categories contained relatively small numbers of participants, resulting in wider CIs for some estimates. Despite these limitations, the study provides useful UAE-specific data on overweight/obesity and its observed associations with behavioral and familial factors. Future population-based and longitudinal studies using validated behavioral measures and sampling methods that account for school-level clustering could further evaluate these associations and improve generalizability.

## Conclusions

This study found that overweight and obesity were common in this sample of adolescents in the UAE. Male gender, a family history of obesity, frequently eating when not hungry or bored, and reporting no participation in at least 60 minutes of out-of-school moderate-to-vigorous physical activity on any day of the week were significantly associated with overweight or obesity in the multivariable analysis. The primary hypothesis was not supported, as recreational screen time was not associated with overweight/obesity in the regression analysis. Although these findings are broadly consistent with evidence from other populations, this study adds UAE-specific evidence on the observed associations between these factors and adolescent overweight and obesity.

These findings highlight the importance of comprehensive public health strategies that promote regular physical activity and healthy eating behaviors among adolescents. Schools, families, and healthcare providers can play an important role in supporting healthy lifestyle habits and creating environments that encourage healthy weight from an early age. As this was a cross-sectional study, the observed associations should not be interpreted as causal, and temporality between the identified factors and overweight or obesity cannot be established. Further longitudinal and intervention studies are needed to better understand these relationships and to guide evidence-based obesity prevention strategies in the UAE.

## Appendices

Questionnaire structure and source note

This appendix presents the self-administered questionnaire used in the study, with the wording and response options of each item. It has six sections: background information, medical history, family history, nutrition, exercise and sleep habits, and screen time.

The questionnaire was developed by the research team based on the study objectives and published literature, and the physical activity and screen time items followed WHO recommendations [6]. A panel of consultant pediatricians from Thumbay University Hospital assessed its content and face validity, and items were revised accordingly. Formal reliability testing was not performed. Response options combined for analysis (e.g., "two hours" and "three hours" as "two to three hours") are described in the Materials and Methods section.

Source note: Study-developed questionnaire by Sana Fatima, Lena Sayed Taha, Sarah Sayed Taha, Ibraheem Mohammed, Abdallah Jaber, and Shatha AlSharbatti.

Questionnaire

**Table 8:** Section A: Background Information

No.	Question	Response options
1	How old are you?	14 years; 15 years; 16 years; 17 years; 18 years
2	Gender	Female; male
3	Where did you spend most of your education?	Private schools; government schools

**Table 9:** Section B: Personal and Family Medical History

No.	Question	Response options
4	Do you have any chronic diseases (e.g., diabetes, asthma, autoimmune disorders)?	Yes; no
5	Do you have any allergies (food, medications, pets, others)? If yes, please specify.	Yes (please specify); no
6	Does anyone in your immediate family (mother, father, sister, brother) have any of the following? (Check all that apply)	Type 1 diabetes; type 2 diabetes; obesity; stroke; liver disease; hypothyroidism; high cholesterol levels; hypertension; chronic heart disease; asthma; sleep apnea; none of the above

**Table 10:** Section C: Nutrition

No.	Question	Response options
7	On a typical day, how many meals do you eat?	1 meal; 2 meals; 3 meals; 4 meals; 5 meals or more
8	In a typical week, how many servings (one piece) of fruit/vegetables do you eat?	None; 1 serving; 2 servings; 3 servings or more
9	In a typical week, how many times do you drink soda or other sweetened beverages?	None; 1 time; 2 times; 3 times or more
10	How many liters of water do you drink per day?	0.5 L; 1 L; 1.5 L; more than 1.5 L
11	In a typical week, how many servings of snacks (cupcakes, cookies, chips, crackers, ice cream, candy, or doughnuts) do you eat?	None; 1 serving; 2 servings; 3 servings or more
12	How many times per week do you eat fast food?	Never; 1 time; 2 times; 3-5 times; more than 5 times
13	How often do you eat from the school cafeteria?	Every day; once a week; 2 times a week; 3 times a week; never
14	How often do you bring packed lunches to school?	Every day; once a week; 2 times a week; 3 times a week; never
15	Do you eat when you are not hungry or feeling bored?	Never; rarely; sometimes; frequently

**Table 11:** Section D: Exercise and Sleep Habits

No.	Question	Response options
16	How many days per week do you participate in out-of-school physical activity of moderate-to-vigorous intensity for at least 60 minutes per day?	None; 1 day; 2 days; 3 days; 4 days; 5 days
17	In an average week when you are in school, on how many days do you go to physical education (PE) classes?	0 days; 1 day; 2 days; 3 days; 4 days; 5 days
18	How many hours of sleep do you get on a weekend night?	6 hours or less; 7-8 hours; 9-10 hours; more than 10 hours
19	How many hours of sleep do you get on a school night?	6 hours or less; 7-8 hours; 9-10 hours; more than 10 hours

**Table 12:** Section E: Devices and Screen Times

No.	Question	Response options
20	Please indicate the devices you own. (Check all that apply)	Cellular phone (non-smartphone); smartphone; tablet (e.g., iPad, Kindle, Google Nexus); video game console (e.g., Xbox, Wii); computer
21	Do you have any of the following in your bedroom? (TV, computer, laptop, tablet, video games, etc.)	Yes; no
22	How many hours do you typically spend on a screen for school-related activities (homework, school papers, school projects, etc.)?	None; 1 hour or less; 2-3 hours; 4-5 hours; more than 5 hours
23	On a school day, how many hours do you typically spend on a screen, such as watching television, playing video games, or using a computer or phone (not including active screen-based games that involve physical movement)?	None; 1 hour or less; 2-3 hours; 4-5 hours; more than 5 hours
24	On a weekend day, how many hours do you typically spend on a screen, such as watching television, playing video games, or using a computer or phone (not including active screen-based games that involve physical movement)?	None; 1 hour or less; 2-3 hours; 4-5 hours; more than 5 hours
25	Do your parents/guardians limit your non-educational screen time?	Yes; No
26	On average, how many hours per day do you engage with a friend using electronic entertainment devices (e.g., playing or chatting on WhatsApp)?	None; 1 hour or less; 2-3 hours; 4-5 hours; more than 5 hours
27	How often do you eat while watching something (TV, YouTube, etc.)?	Never; hardly ever; sometimes; often; always
28	How often do you use social media?	I do not use social media; a few times a month; about once a week; a few times a week; about once a day; several times a day; about once an hour; more than once an hour
